# The Health Cost of Transport in Cities

**DOI:** 10.1007/s40572-021-00308-6

**Published:** 2021-03-08

**Authors:** Stefan Gössling, Jessica Nicolosi, Todd Litman

**Affiliations:** 1 School of Business and Economics, Linnaeus University, 391 82 Kalmar, Sweden; 2 Service Management and Service Studies, Lund University, Box 882, 25108 Helsingborg, Sweden; 3 T3 Freiburg, Clayallee 177, 14195 Berlin, Germany; 4 Victoria Transport Policy Institute, 1250 Rudlin Street, Victoria, BC V8V 3R7 Canada

**Keywords:** Active transport, Cities, Cost-benefit analysis, Cycling, Transport policy, Urban planning

## Abstract

**Purpose of Review:**

The study aims to provide an understanding of health cost assessments of different transport modes in urban contexts, and their relevance for transport planning and political decision-making.

**Recent Findings:**

There is strong evidence that motorized transportation imposes a high health cost on society, and specifically children. In contrast, active transport is a very significant health benefit.

**Summary:**

Economic analyses support urban change in favor of compact neighborhoods and public transit, as well as infrastructure exclusively devoted to active transport. Private cars need to be restricted because of the high cost they impose on society.

## Introduction

This review summarizes recent advances in transport economics with relevance for health cost assessments in urban contexts. It discusses the role of cost-benefit and cost-utility analyses in transport decision-making, as well as the most relevant insights gained from a wide range of studies. This provides the basis for a conceptualization of a comprehensive transport health cost model that considers both physical and mental health aspects. A comparison of the role of car and active transport (walking and cycling) is presented, and key insights for transport planners are discussed. The paper also highlights knowledge gaps.

## Current Knowledge on the Topic

Cost-benefit analyses (CBA) as well as cost-utility analyses (CUA) of transport systems and (planned) interventions have gained importance for transport politics and planning [8, 16, 18, 66]. These analyses have various functions, such as to determine whether a transport infrastructure investment is economically meaningful, to rank alternative infrastructure investment options, to compare the cost/benefit of different transport modes, to economically assess the outcomes of planned or completed interventions such as new bicycle tracks, or to understand economic implications of transport systems more generally.

Cost-benefit analyses reduce complex social and environmental processes to monetary value, and they are necessarily reductionist. Results will depend on the choice of parameters included, their factor cost, and the time horizons over which analyses are integrated. The use of cost analyses is weakened by the absence of market values for specific parameters, value incommensurability, risks of double counting, and fairness issues [5, 29].

Transport cost analyses have focused on a wide range of aspects, such as the social cost of transport systems at different scales [22, 50]; the cost of specific transport infrastructure developments [41]; the value of physically active transport [47]; cost comparisons of transport modes [63]; transport mode substitution [60]; transport system change [62]; and monetarization of individual cost aspects, such as air pollutants, noise, or traffic risks [23]. Health aspects have a key role in any transport cost assessments, but are often overlooked or undervalued [45].

### Total Health Cost of Urban Transport

**Fig. 1:**
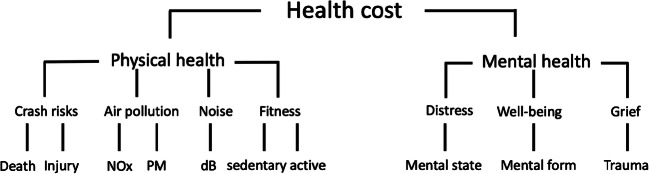
Total transport health cost

Transport health cost assessments are complex, because of the many parameters involved, their monetarization, and interaction [31, 36, 40]. Figure 1 conceptualizes total transport health cost, distinguishing physical and mental health dimensions. Costs are related to crash risks, air pollution, noise, sedentary/active lifestyles (physical health), and distress, well-being, and grief (mental health). Physical and mental health are often interdependent, and their cost can accrue to the individual or society. Unit costs can be higher in urban contexts, where a larger population is exposed to impacts related to more densely built environments, including congestion, noise, and air pollution. Higher traffic crash rates, obesity, and sedentary living are generally more prevalent in rural contexts.

The definition of parameters to be included in cost assessments has relevance for results, and should be as comprehensive as possible. Figure 1 includes *crash risks*, i.e., road accidents resulting in death and injury, longer-term injury outcomes (disabilities), or pain. *Air pollution* refers to the effects of exhaust from tailpipes (specifically nitrous oxides, NO_x_), as well as pollutant related to exhaust, break and tire wear (particulate matter, PM) [40]. *Noise* comprises unwanted noise, vibrations, and infrasound (“decibel,” dB, in Fig. 1), which have been linked to morbidity and mortality, as well as the cognitive impairment of children [3, 71]. *Fitness* refers to lifestyles, which may be more sedentary if car-based, or more active if involving cycling or walking. Lack of physical activity is linked to morbidity and mortality; physically active lifestyles have been shown to result in reduced obesity as well as greater life expectation, and hence a decline in all-cause mortality [7, 14, 24, 34, 59].

In the mental health categories, *mental state* (distress) is situational and can be related to (perceived) safety risks, negative experiences including harassment, or exhaust smell [4, 23, 30]. *Mental form* is a more permanent condition of feeling well that is influenced by urban design and mode shares. Secure cycling or walking infrastructure, built environments with green spaces, for example, affect subjective well-being [1, 42, 64]. Active transport also improves cognitive functioning and psychological well-being (self-esteem, positive mood), with a corresponding decline in mental health problems [43, 58, 70]. *Grief* (trauma) can result out of injuries or loss of loved ones in crashes, including animals [12, 33, 37].

Health impacts are usually averaged, even though they increase with traffic density, depend on the time of the day and the specific transport mode, while imposing different risks on different traffic and non-traffic user groups [18, 41, 52]. For example, noise levels vary between motorcycles, trucks, and car models, while crash risks are greater for vehicles with greater mass [48, 49]. There is much evidence that health impacts are not evenly imposed on society: children, as well as active transport users, are significantly more affected by air pollutants, for example [2, 17]. Elderly people are more exposed to injury risks [9], and pedestrians more than cyclists [48]. Teenage and young vehicle drivers pose the greatest traffic risk per kilometer of travel [72], followed by elderly drivers [68]. Road crashes are the leading reason for death among 5–29 year olds [72], and increase in areas where people travel more by motor vehicles. Bicyclists face significantly higher injury risks than car occupants [57], but they are less often responsible for crashes, at least in countries where cycling is less common [44].

Table 1 provides an overview of the economic methods used to assess health cost parameters.

**Table 1:** Methodologies to assess health cost items

Aspect	Methodology	Reference
Crashes	Damage cost approach (WTP) Value of a statistical life	European Commission [18]
Air pollution	Health service/damage cost Contingent valuation (WTP) Value of a statistical life Mortality risk reduction (DALYs)	Danish Ministry of Transport [13] Götschi et al. [25] Rodrigues et al. (2020)
Noise	Hedonic pricing/stated preference (WTP)	Danish Ministry of Transport [13]
Fitness	Productivity loss Health service cost Value of a statistical life	COWI [11] Götschi et al. [25]
Stress	Hedonic pricing (WTP) MET h/year	Cohen et al. [10]
Well-being	MET h/year	Cohen et al. [10]
Grief	Hedonic pricing (WTP)	Miller [51]

*WTP* willingness-to-pay, *WTA* willingness-to-accept, *DALY* disability-adjusted life years, *MET* metabolic equivalent of task

It is beyond the scope of this review to go through all methods; the table serves the purpose of illustrating the diverse range of approaches to economic evaluation. While impacts are usually assessed separately, they are sometimes associated: a traffic injury is likely to have both a physical and mental health impact, for example. This poses a risk of double-counting, though more often, mental health aspects remain unaccounted for. There is also a likelihood of unrecognized feedback-loops. For example, involvement in an accident can result in insomnia, while sleep disorders would again increase traffic risks. As most health cost models only consider a limited number of parameters (i.e., ignoring in particular mental health) while not fully accounting for active health benefits [25, 45, 65], a central conclusion is that most assessments underestimate the *cost* of motorized transportation and the *benefits* of active transport.

Various platforms are now available as tools for assessments. For instance, the World Health Organization’s Health Economic Assessment Tool (HEAT) can be used to support investment decisions in active travel [25]. National tools exist as well (e.g., [8] (Denmark); [15] (Australia); Department of Transport [16]; [55, 66] (USA)). Tools provide order of magnitude estimates, for example, for changes in walking/cycling levels at different scales (national, city, project), and with attention paid to health impacts (mortality due to exposure to air pollution, crash risks, and physical activity). A general problem is that while some aspects (medical expenses and disability compensation) can be monetized, this is very difficult for grief, loss of quality of life, and premature deaths.

### Economic Evidence in Favor of Active Mobility

A large number of recent publications have focused on the economic assessment of cycling or walking. These studies assess the health implications of transport behavior changes as a result of infrastructure investments that increase speed, (perceived) safety, and accessibility for active transport users [25, 74]. The outcome of physical activity is a reduction in morbidity and mortality [21, 52, 53]. Even where active transport health benefits have to be weighed against additional risks (air pollution, crashes), assessments suggest that benefits outweigh harms [14, 20, 74].

Available studies are integrated over different time horizons and use different parameters and unit costs. Yet, findings unambiguously support that any substitution of individual motorized for active transport will yield significant economic benefits. Investments in cycling or walking infrastructure have repay periods of between 1 and 10 years [26, 67]. These studies only consider benefits of physical activity, along with reduced crash risks and air pollution [52], and leave a wide range of health aspects unaccounted for (Fig. 1). Active travel also reduces transport greenhouse gas emissions significantly, and thus reduces health cost even indirectly [6].

To provide some examples of cost assessments, the World Bank [69] estimates that halving road traffic injuries in China, India, the Philippines, Tanzania, and Thailand will yield welfare benefits equivalent to 6–32% of national GDP. In another study of US commuters in metropolitan areas, Grabow et al. [26] conclude that a scenario of half the population cycling rather than driving to work yields a benefit of US$1900 per person and year, corresponding to a 5% reduction in healthcare cost. Likewise, a daily exercise of cycling just 3.4 km in urban areas of England and Wales will reduce the cost of healthcare by 0.8% [32]. In Sweden, the net benefit from a 15% increase in the number of bicycle commuters in Stockholm was estimated at 8.7% of the municipality’s healthcare budget (3.7% after discounting) [39]. An investment in sidewalks in a Wisconsin community suggests a cost-benefit ratio of 1.81 [27]. Cycling infrastructure expansion also leads to significant gains in quality-adjusted life years, and is thus highly cost-effective even in high-income economies [41]. Even small changes to urban designs, such as to close down roads for through motor traffic, can lead to very significant health benefits [1].

## Conclusions

Irrespective of scale of analysis, studies suggest that motorized individual transport represents a cost imposed on society that is not covered by fees and taxes. In contrast, active transport constitutes a benefit to both individual and society, mostly because of positive health outcomes. Health effects are highly relevant in cities, where traffic density is higher, exposing larger populations to congestion, air pollution, noise, or crash risks. Health impacts are potentially very large, but often overlooked (Todd 2013). This tends to overvalue car-oriented planning and sprawl, while undervaluing active and public transport [19, 28].

Given the health benefits of “15-min” cities, in which all daily urban necessities can be reached on foot or by bicycle, compact neighborhoods should be urban development priorities. Cycling in particular is a fast and convenient alternative [61], while walking benefits from connectivity ([38]). Urban planners should also seek to restrict and limit motorized forms of transportation, specifically the private car [73]. The attractiveness of active mobility will increase where active transport is separated from motorized transport, as travelers value quiet roads, engaging in significant detours to avoid traffic risks, noise, and exhaust [23, 54]. As cities such as Copenhagen or Amsterdam have cycle trip percentages exceeding 30% of all trips [73], urban re-design should be thought in bold terms [35, 56]. Comprehensive economic analyses will make it easier to justify investments [46]. However, infrastructure change does not have to be costly, if urban re-design focuses on the development of entire networks of roads devoted solely to active transport, or the conversion of entire city blocks into largely car-free neighborhoods. Micromobility streets, also dubbed “happy streets,” or superblocks as implemented in Barcelona reduce risks, pollution, and noise, while creating more livable urban environments with significant mental health benefits [56].

The review also identified various areas that require further research. In economic terms, many of the categories in the total health cost framework are inadequately monetarized. A better understanding of mental cost aspects, for example, is likely to further support the case for investments in active mobility. There is also a need to better understand the cost and benefits of specific transport modes, such as motorcycles, trucks, public transport, and e-mobility. It remains unclear how e-bikes and e-scooters compare in economic health terms to physically active forms of mobility. These issues require further clarification.

## References

[1] Aldred R, Croft J (2019). Evaluating active travel and health economic impacts of small streetscape schemes: an exploratory study in London. J Transp Health.

[2] Alotaibi R, Bechle M, Marshall JD, Ramani T, Zietsman J, Nieuwenhuijsen MJ, Khreis H (2019). Traffic related air pollution and the burden of childhood asthma in the contiguous United States in 2000 and 2010. Environ Int.

[3] Babisch W (2015). The cardiovascular effects of noise on man. J Acoust Soc Am.

[4] Bissell D (2010). Passenger mobilities: affective atmospheres and the sociality of public transport. Environ Plan D: Soc Space.

[5] Bithas K (2011). Sustainability and externalities: is the internalization of externalities a sufficient condition for sustainability?. Ecol Econ.

[6] Brand C, Goodman A, Ogilvie D, iConnect Consortium (2014). Evaluating the impacts of new walking and cycling infrastructure on carbon dioxide emissions from motorized travel: a controlled longitudinal study. Appl Energy.

[7] Celis-Morales CA, Lyall DM, Welsh P, Anderson J, Steell L, Guo Y, Gill JM (2017). Association between active commuting and incident cardiovascular disease, cancer, and mortality: prospective cohort study. BMJ.

[8] Center for Transport Analytics (2019). Transportøkonomiske Enhedspriser. Available: https://www.cta.man.dtu.dk/modelbibliotek/teresa/transportoekonomiske-enhedspriser. Accessed 23 January 2021

[9] Chong S, Poulos R, Olivier J, Watson WL, Grzebieta R (2010). Relative injury severity among vulnerable non-motorised road users: comparative analysis of injury arising from bicycle–motor vehicle and bicycle–pedestrian collisions. Accid Anal Prev.

[10] Cohen DA, Marsh T, Williamson S, Golinelli D, McKenzie TL (2012). Impact and cost-effectiveness of family fitness zones: a natural experiment in urban public parks. Health Place.

[11] COWI (2009). Samfundsøkonomiske Analyser af Cykeltiltag - Metode og Cases. Available: www.kk.dk. Accessed 24 January 2021

[12] Crawford BA, Andrews KM (2016). Drivers’ attitudes toward wildlife-vehicle collisions with reptiles and other taxa. Anim Conserv.

[13] Danish Ministry of Transport (2004). External costs of transport. Copenhagen: Danish Ministry of Transport. Available: https://transportministeriet.dk/media/2005/1streport.pdf. Accessed 4 February 2021

[14] De Hartog JJ, Boogaard H, Nijland H, Hoek G (2010). Do the health benefits of cycling outweigh the risks?. Environ Health Perspect.

[15] Department of Infrastructure and Regional Development (2017). Australian transport assessment and planning guidelines. Available: https://www.atap.gov.au/. Accessed 4 February 2021

[16] Department of Transport (2018). Investing in cycling and walking: the economic case for action. Available: https://www.gov.uk/government/publications/cycling-and-walking-the-economic-case-for-action. Accessed 22 January 2021

[17] deSouza P, Lu R, Kinney P, & Zheng S (2020). Exposures to multiple air pollutants while commuting: evidence from Zhengzhou, China. Atmos Environ, 118168.

[18] European Commission (2019). Handbook on the external costs of transport. European Commission: Brussels. Available: https://www.cedelft.eu/en/publications/2311/handbook-on-the-external-costs-of-transport-version-2019. Accessed 4 February 2021

[19] Ewing R, Schmid T, Killingsworth R, Zlot A, Raudenbush S (2003). Relationship between urban sprawl and physical activity, obesity, and morbidity. Am J Health Promot.

[20] Fishman E, Schepers P, Kamphuis CBM (2015). Dutch cycling: quantifying the health and related economic benefits. Am J Public Health.

[21] Fletcher GF, Landolfo C, Niebauer J, Ozemek C, Arena R, Lavie CJ (2018). Promoting physical activity and exercise: JACC Health Promotion Series. J Am Coll Cardiol.

[22] Gössling S, Choi A, Dekker K, Metzler D (2019). The social cost of automobility, cycling and walking in the European Union. Ecol Econ.

[23] Gössling S, Litman T, Humpe A, Metzler D (2019). Effects of perceived traffic risks, noise, and exhaust smells on bicyclist behaviour: an economic evaluation. Sustainability.

[24] Gotschi T (2011). Costs and benefits of bicycling investments in Portland, Oregon. J Phys Act Health.

[25] Götschi T, Kahlmeier S, Castro A, Brand C, Cavill N, Kelly P, Lieb C, Rojas-Rueda D, Woodcock J, Racioppi F (2020). Integrated impact assessment of active travel: expanding the scope of the Health Economic Assessment Tool (HEAT) for walking and cycling. Int J Environ Res Public Health.

[26] Grabow ML, Spak SN, Holloway T, Stone B, Mednick AC, Patz JA (2012). Air quality and exercise-related health benefits from reduced car travel in the midwestern United States. Environ Health Perspect.

[27] Guo JY, Gandavarapu S (2010). An economic evaluation of health-promotive built environment changes. Prev Med.

[28] Hamidi S, Ewing R, Tatalovich Z, Grace JB, Berrigan D (2018). Associations between urban sprawl and life expectancy in the United States. Int J Environ Res Public Health.

[29] Hanley N, Spash C (1993). Cost benefit analysis and the environment Edward Elgar.

[30] Heesch KC, Sahlqvist S, Garrard J (2011). Cyclists’ experiences of harassment from motorists: findings from a survey of cyclists in Queensland, Australia. Prev Med.

[31] Hoek G, Brunekreef B, Goldbohm S, Fischer P, van den Brandt PA (2002). Association between mortality and indicators of traffic-related air pollution in the Netherlands: A Cohort Study. Lancet.

[32] Jarrett J, Woodcock J, Griffiths UK, Chalabi Z, Edwards P, Roberts I, Haines A (2012). Effect of increasing active travel in urban England and Wales on costs to the national health service. Lancet.

[33] Kaufman KR, Kaufman ND (2006). And then the dog died. Death stud.

[34] Kelly P, Kahlmeier S, Götschi T, Orsini N, Richards J, Roberts N (2014). Systematic review and meta-analysis of reduction in all-cause mortality from walking and cycling and shape of dose response relationship. Int J Behav Nutr Phys Act.

[35] Khreis H, Cirach M, Mueller N, de Hoogh K, Hoek G, Nieuwenhuijsen MJ, Rojas-Rueda D (2019). Outdoor air pollution and the burden of childhood asthma across Europe. Eur Respir J.

[36] Klæboe R, Kolbenstvedt M, Clench-Aas J, Bartonova A (2000). Oslo traffic study—Part 1: an integrated approach to assess the combined effects of noise and air pollution on annoyance. Atmos Environ.

[37] Kolata RJ, Johnston DE (1975). Motor vehicle accidents in urban dogs: a study of 600 cases. J Am Vet Med Assoc.

[38] Koohsari MJ, Sugiyama T, Lamb KE, Villanueva K, Owen N (2014). Street connectivity and walking for transport: role of neighborhood destinations. Prev Med.

[39] Kriit HK, Williams JS, Lindholm L, Forsberg B, Sommar JN (2019). Health economic assessment of a scenario to promote bicycling as active transport in Stockholm, Sweden. BMJ Open.

[40] Künzli N, Kaiser R, Medina S, Studnicka M, Chanel O, Filliger P, Herry M, Horak F, Puybonnieux-Texier V, Quénel P, Schneider J, Seethaler R, Vergnaud JC, Sommer H (2000). Public-health impact of outdoor and traffic-related air pollution: a European assessment. Lancet.

[41] Lamu AN, Jbaily A, Verguet S, Robberstad B, Norheim OF (2020). Is Cycle network expansion cost-effective? A health economic evaluation of cycling in Oslo. BMC Public Health.

[42] Lee AC, Maheswaran R (2011). The health benefits of urban green spaces: a review of the evidence. J Public Health.

[43] Leyland LA, Spencer B, Beale N, Jones T, Van Reekum CM (2019). The effect of cycling on cognitive function and well-being in older adults. PLoS One.

[44] Lindsay, V. L. (2013). Injured cyclist profile: an in-depth study of a sample of cyclists injured in road crashes in South Australia (No. CASR112). Centre for Automotive Safety Research, University of Adelaide.

[45] Litman T (2013). Transportation and public health. Annu Rev Public Health.

[46] Litman T (2018). Toward more comprehensive evaluation of traffic risks and safety strategies. Res Transp Bus Manag.

[47] Litman TA (2003). Economic value of walkability. Transp Res Rec.

[48] Maki T, Kajzer J, Mizuno K, Sekine Y (2003). Comparative analysis of vehicle–bicyclist and vehicle–pedestrian accidents in Japan. Accid Anal Prev.

[49] Malczyk A. Das Unfallgeschehen mit SUV in Deutschland. VKU Verkehrsunfall und Fahrzeugtechnik. 2012;50(10).

[50] Meschik M (2012). Reshaping city traffic towards sustainability why transport policy should favor the bicycle instead of car traffic. Procedia Soc Behav Sci.

[51] Miller, T., Viner, J., Rossman, S., Pindus, N., Gellert, W., Douglass, J., ... & Blomquist, G. (1991). The costs of highway crashes (No. FHWA-RD-91-055). United States. Federal Highway Administration.

[52] Mizdrak A, Blakely T, Cleghorn CL, Cobiac LJ (2019). Potential of active transport to improve health, reduce healthcare costs, and reduce greenhouse gas emissions: a modelling study. PLoS One.

[53] Mueller N, Rojas-Rueda D, Cole-Hunter T, De Nazelle A, Dons E, Gerike R (2015). Health impact assessment of active transportation: a systematic review. Prev Med.

[54] Mulley C, Tyson R, McCue P, Rissel C, Munro C (2013). Valuing active travel: including the health benefits of sustainable transport in transportation appraisal frameworks. Res Transp Bus Manag.

[55] New Zealand Transport Agency (2020). Economic evaluation manual. Available: https://wwwnztagovtnz/resources/monetised-benefits-and-costs-manual/ Accessed 27 January 2021.

[56] Nieuwenhuijsen MJ (2020). Urban and Transport planning pathways to carbon neutral, liveable and healthy cities: a review of the current evidence. Environ Int, 105661. 10.1016/j.envint.2020.105661 32307209

[57] Nilsson P, Stigson H, Ohlin M, Strandroth J (2017). Modelling the effect on injuries and fatalities when changing mode of transport from car to bicycle. Accid Anal Prev.

[58] Pretty J, Peacock J, Hine R, Sellens M, South N, Griffin M (2007). Green exercise in the UK countryside: effects on health and psychological well-being, and implications for policy and planning. J Environ Plan Manag.

[59] Pucher J, Buehler R, Bassett DR, Dannenberg AL (2010). Walking and cycling to health: a comparative analysis of city, state, and international data. Am J Public Health.

[60] Rabl A, De Nazelle A (2012). Benefits of shift from car to active transport. Transp Policy.

[61] Raustorp J, Koglin T (2019). The potential for active commuting by bicycle and its possible effects on public health. J Transp Health.

[62] Rodrigues PF, Alvim-Ferraz MCM, Martins, FG, Saldiva, P, Sá TH, Sousa SIV. Health economic assessment of a shift to active transport. Environ Pollut. 2020;258:113745. 10.1016/j.envpol.2019.113745 31855678

[63] Saighani A, Sommer C (2019). Method for an economical assessment of urban transport systems. Transp Res Procedia.

[64] Singleton PA (2019). Walking (and cycling) to well-being: modal and other determinants of subjective well-being during the commute. Travel Behav Soc.

[65] Standen C, Greaves S, Collins AT, Crane M, Rissel C (2019). The value of slow travel: economic appraisal of cycling projects using the logsum measure of consumer surplus. Transp Res A Policy Pract.

[66] State of California (2020). Integrated transport and health impact model. Available: https://skylab.cdph.ca.gov/HealthyMobilityOptionTool-ITHIM/. Accessed 22 January 2021.

[67] Taddei C, Gnesotto R, Forni S, Bonaccorsi G, Vannucci A, Garofalo G (2015). Cycling promotion and non-communicable disease prevention: health impact assessment and economic evaluation of cycling to work or school in Florence. PLoS One.

[68] Tefft BC (2008). Risks older drivers pose to themselves and to other road users. J Saf Res.

[69] The World Bank (2017). The high toll of traffic injuries: unacceptable and preventable. Available: https://www.worldbank.org/en/programs/global-road-safety-facility/publication/the-high-toll-of-traffic-injuries-unacceptable-and-preventable. Accessed 24 January 2021.

[70] Wells NM (2000). At home with nature: effects of “greenness” on children’s cognitive functioning. Environ Behav.

[71] WHO (2011). Burden of disease from environmental noise: quantification of healthy life years lost in Europe. World Health Organization. Regional Office for Europe. Available: https://apps.who.int/iris/handle/10665/326424. Accessed 24 January 2021.

[72] World Health Organization. (2018). Global status report on road safety 2018: Summary (No. WHO/NMH/NVI/18.20). World Health Organization.

[73] World Health Organization. (2020). Cyclist safety: an information resource for decision-makers and practitioners.

[74] Woodcock J, Tainio M, Cheshire J, O’Brien O, Goodman A. Health effects of the London bicycle sharing system: Health Impact Modelling Study. BMJ. 2014;348. 10.1136/bmj.g425 PMC3923979 24524928

